# The effect of BMI on long-term outcome in patients with rectal cancer and establishment of a nomogram prediction model

**DOI:** 10.1186/s12876-023-02638-1

**Published:** 2023-01-09

**Authors:** Yang Zhang, Xuyang Yang, Zixuan Zhuang, Mingtian Wei, Wenjian Meng, Xiangbing Deng, Ziqiang Wang

**Affiliations:** Colorectal Cancer Center, Department of General Surgery, West China Hospital, Sichuan University, No. 37 Guo Xue Alley, Chengdu, 610041 China

**Keywords:** Rectal cancer, Body mass index, Long-term outcome, Nomogram

## Abstract

**Background:**

The effects of body mass index (BMI) in patients with rectal cancer have been poorly studied and are still controversial. In this study, we aimed to assess the effect of BMI on the long-term outcome in patients with rectal cancer after radical surgery.

**Materials and methods:**

Between April 2012 and December 2020, patients who received total mesorectal excision (TME) surgery were enrolled in the study. Patients were divided into four groups according to BMI level. Kaplan–Meier survival curves with log-rank tests were used to analyze overall survival (OS), Disease-free survival (DFS), local recurrence-free survival and distant metastasis-free survival. Univariate and multivariate analyses were performed to identify the risk factors associated with the long-term outcome. Nomograms were developed to predict the OS and DFS based on independent prognostic factors.

**Results:**

A total of 688 patients were included in this study. The median follow-up time was 69 months. The 5-year OS rates of the control, underweight, overweight and obese groups were 79.2%, 62.2%, 88.7% and 86.3%, respectively. The 5-year DFS rates were 74.8%, 58.2%, 80.5% and 81.4%, respectively. Overweight (HR 0.534; 95% CI 0.332–0.860, *p* = 0.010) was an independent protective factor for OS and DFS (HR 0.675; 95% CI 0.461–0.989, *p* = 0.044). Underweight was an independent risk factor for DFS (HR = 1.623; 95% CI 1.034–2.548; *p* = 0.035), and had a trend to be an independent risk factor for OS (HR 1.594; 95% 0.954–2.663; *p* = 0.075). Nomograms were established to predict the 2-year OS, 5-year OS, 2-year DFS and 5-year DFS with an area under curve (AUC) of 0.767, 0.712, 0.746 and 0.734, respectively.

**Conclusions:**

For rectal cancer patients after radical surgery, overweight was an independent protective factor for OS and DFS. Underweight was an independent risk factor for DFS and had a trend to be an independent risk factor for OS. Nomograms incorporating BMI and other prognostic factors could be helpful to predict long-term outcome.

**Supplementary Information:**

The online version contains supplementary material available at 10.1186/s12876-023-02638-1.

## Introduction

Rectal cancer is one of the most prevalent cancers worldwide and seriously threatens human health Sung, Ferlay, Siegel, Laversanne, Soerjomataram, Jemal and Bray [1]. In recent years, with the establishment of standard total mesorectal excision (TME) and the wide application of neoadjuvant chemoradiotherapy (nCRT), the prognosis of rectal cancer patients was greatly improved [2, 3]. Numerous studies have tried to explore prognostic factors associated with rectal cancer. These existing studies mainly focused on the prognosis of the tumor pathology, treatment mode and some molecular markers [4–7]. However, the effects of some demographic or biological characteristics, such as body mass index (BMI), have been poorly studied and are still controversial.

Some studies have reported that increased BMI leads to worse outcomes of rectal cancer [8–11]. These studies mainly revealed that increased BMI or obesity was associated with a higher local recurrence (LR) rate [10, 11]. However, another study demonstrated that the OS of overweight/obese patients was significantly better than that of underweight/normal-weight patients [12]. Meanwhile, other studies reported that lower BMI or underweight was associated with poorer overall survival (OS) [13–15]. Moreover, different from the above studies, another two studies revealed that both low BMI and high BMI could lead to worse survival of rectal cancer patients when compared to normal BMI [16, 17]. Therefore, the impact of BMI on the oncologic outcome of rectal cancer patients remains disputed based on the published literature.

As living standards are improving, the population of overweight/obese people has increased dramatically. On the other hand, malnutrition commonly occurs during nCRT in cancer patients. Therefore, this means that colorectal surgeons will face more patients with abnormal BMI. Hence, further research is necessary to assess the impact of BMI on the prognosis of rectal cancer patients.

In the current study, we aimed to assess the effect of BMI on the long-term outcome of rectal cancer after radical surgery.

## Materials and methods

### Patients and data collection

This was a single-center retrospective cohort study. This study conforms to the STROBE guideline (see “Additional file 1: STROBE Checklist”) [18]. Between April 2012 and December 2020, patients who received TME surgery were enrolled in the study. The eligible criteria were rectal cancer proven by pathological biopsy and curative TME surgery. The exclusion criteria included tumor distance from the anal verge (AV) > 12 cm, recurrent rectal cancer, multiple primary cancers or simultaneous distant metastasis, previous treatment for other cancers and incomplete medical records.

The following information was collected: sex, age, BMI, tumor distance from AV, clinical T stage (cT), clinical N stage (cN), neoadjuvant therapy (nCRT), adjuvant therapy, type of surgery (sphincter-preserving/nonsphincter-preserving), pathological T stage (pT), pathological N stage (pN), and the number of mesorectal lymph nodes harvested. The clinical staging was assessed based on abdominal magnetic resonance imaging (MRI) combined with enhanced computed tomography (CT). TNM staging was based on the UICC’s TNM staging system [19]. BMI was defined as weight divided by height squared (kg/m^2^). The BMI of patients was measured on the day before surgery. Patients were divided into four groups according to the BMI level proposed by the World Health Organization (WHO) [20]. In detail, underweight was defined as BMI < 18.5 kg/m^2^, normal (control) 18.5 to < 25.0 kg/m^2^, overweight 25.0 to < 30.0 kg/m^2^, and BMI ≥ 30 kg/m^2^ was considered obese.

### Surveillance

The follow-up strategy was consistent with our previous study [21]. In short, physical examination, including digital rectal examination and serum tumor markers, was conducted every 3 months in the first 2 years, every 6 months in the next 3 years and every year after 5 years. For the first two years, it is recommended that chest CT and abdominal CT enhancement scans be performed every 6 months and once a year thereafter.

The primary endpoints of this study were OS and disease-free survival (DFS). OS was defined as the time from the end of surgery to the occurrence of death or the final follow-up time. DFS was defined as the time between surgery and local recurrence or distant metastasis or the final follow-up time. The secondary endpoints included local recurrence-free survival and distant metastasis-free survival. The time from the end of surgery to local recurrence or to the final follow-up time was considered the local recurrence-free survival. Distant metastasis-free survival was defined as the time from the end of surgery to distant metastasis-free survival or the final follow-up time.

### Statistical analysis

All statistical analyses were conducted by R software (R software, version 4.0.5) and SPSS software version 22.0 (IBM Inc., Armonk, NY, USA). Continuous variables were expressed as the mean (standard deviation), and differences between the two groups were tested using independent-sample *t*-tests. Categorical variables were reported as numbers (percentages), and differences were tested using Pearson’s chi-squared tests or Fisher’s exact tests. Kaplan–Meier survival curves with log-rank tests were used to analyze OS, DFS, local recurrence-free survival and distant metastasis-free survival. Univariate and multivariate analyses were performed to identify the risk factors associated with the long-term outcome. Multivariate analyses were performed using multivariate Cox regression. Nomograms were developed to predict the OS and DFS based on independent prognostic factors. *P* values ≤ 0.05 were considered statistically significant.

## Results

### Patient characteristics

A total of 688 patients were included in this study. According to the BMI classification, there were 447 patients in the control group (BMI 18.5 to < 25 kg/m^2^), 53 patients in the underweight group (BMI < 18.5 kg/m^2^), 171 patients in the overweight group (BMI 25 to < 30 kg/m^2^), and 17 patients in the obese group (BMI ≥ 30 kg/m^2^). The clinicopathological characteristics of the included patients were shown in Table 1. There were no significant differences in baseline variables among the above four groups, except for cT stage (*P* = 0.007) and pT stage (*P* = 0.013). The mean age of all patients was 59.2 years, and the mean tumor distance from the AV was 5.9 cm. A total of 42.4% of the patients received neoadjuvant therapy, including short-course RT (11.3%), long-course CRT (28.8%), and chemotherapy (2.3%). A total of 45.8% of the patients received adjuvant chemotherapy. An average of 11.7 mesorectal lymph nodes were harvested, and 28.3% of all patients developed mesorectal lymph node metastasis proven by pathology. Statistically significant *p* values were shown in bold typeface.

**Table 1:** The clinicopathological characteristics of patients included

Characteristics	Control (BMI 18.5 to < 25 kg/m^2^) n = 447	Underweight (BMI < 18.5 kg/m^2^) n = 53	Overweight (BMI 25 to < 30 kg/m^2^) n = 171	Obese (BMI ≥ 30 kg/m^2^) n = 17	*p* value
Sex, n (%)					0.575
Male	274 (61.3)	28 (52.8)	106 (62.0)	9 (52.9)	
Female	173 (38.7)	25 (47.2)	65 (38.0)	8 (47.1)	
Age, mean ± SD, year	59.0 ± 12.3	60.9 ± 13.2	59.3 ± 11.7	60.8 ± 9.6	0.688
Tumor distance from AV, mean + SD, cm	6.0 ± 2.9	6.0 ± 3.1	5.7 ± 2.8	6.7 ± 2.7	0.343
cT, n (%)					**0.007**
T1	19 (4.3)	0 (0.0)	4 (2.3)	2 (11.8)	0.089
T2	68 (15.2)	9 (17.0)	31 (18.1)	1 (5.9)	0.548
T3	250 (55.9)	29 (54.7)	114 (66.7)	13 (76.5)	**0.038**
T4	110 (24.6)	15 (28.3)	22 (12.9)	1 (5.9)	**0.003**
cN, n (%)					0.462
N0	192 (43.0)	19 (35.8)	71 (41.5)	10 (58.8)	0.404
N1	121 (27.1)	19 (35.8)	54 (31.6)	2 (11.8)	0.173
N2	134 (30.0)	15 (28.3)	46 (26.9)	5 (29.4)	0.900
nCRT, n (%)					0.081
Short-course RT	54 (12.1)	9 (17.0)	14 (8.2)	1 (5.9)	0.249
Long-course CRT	118 (26.4)	19 (35.8)	57 (33.3)	4 (23.5)	0.219
Chemotherapy	8 (1.8)	0 (0.0)	8 (4.7)	0 (0.0)	0.094
Primary surgery	267 (59.7)	25 (47.2)	92 (53.8)	12 (70.6)	0.146
Adjuvant therapy, n (%)	199 (44.5)	28 (52.8)	79 (46.2)	9 (52.9)	0.635
Type of procedure, n(%)					0.171
LAR	327 (73.2)	33 (62.3)	121 (70.8)	14 (82.4)	0.282
ELAPE or Miles	75 (16.8)	13 (24.5)	33 (19.3)	1 (5.9)	0.275
ISR	33 (7.4)	2 (3.8)	13 (26.5)	1 (5.9)	0.791
Hartmann	12 (2.7)	5 (9.4)	4 (2.3)	1 (5.9)	0.055
pT, n (%)					**0.013**
T0	37 (8.3)	2 (3.8)	11 (6.4)	0 (0.0)	0.370
T1	36 (8.1)	1 (1.9)	4 (2.3)	1 (5.9)	**0.032**
T2	124 (27.7)	13 (24.5)	70 (40.9)	7 (41.2)	**0.008**
T3	216 (48.3)	35 (66.0)	78 (45.6)	8 (47.1)	**0.070**
T4	34 (7.6)	2 (3.8)	8 (4.7)	1 (5.9)	0.480
pN, n (%)					0.880
N0	296 (66.2)	36 (67.9)	117 (68.4)	13 (76.5)	0.805
N1	119 (26.6)	12 (22.6)	42 (24.6)	4 (23.5)	0.893
N2	32 (7.2)	5 (9.4)	12 (7.0)	0 (0.0)	0.629
Number of mesorectal lymph nodes harvested, mean ± SD	11.4 ± 7.2	12.9 ± 7.2	12.1 ± 7.2	14.5 ± 9.7	0.138
Number of metastatic lymph nodes in mesorectum, mean ± SD	2.8 ± 2.9	3.1 ± 3.0	3.4 ± 3.7	1.2 ± 0.4	0.390

### Oncological outcomes

The median follow-up time was 69 months. The 5-year OS rates of the control, underweight, overweight and obese groups were 79.2%, 62.2%, 88.7% and 86.3%, respectively. The 5-year DFS rates were 74.8%, 58.2%, 80.5% and 81.4%, respectively. The LR rates were 4.8%, 13.3%, 4.5% and 0%, respectively. The distant metastasis rates of these groups were 29.6%, 33.3%, 20.3% and 18.6%, respectively.

**Fig. 1:**
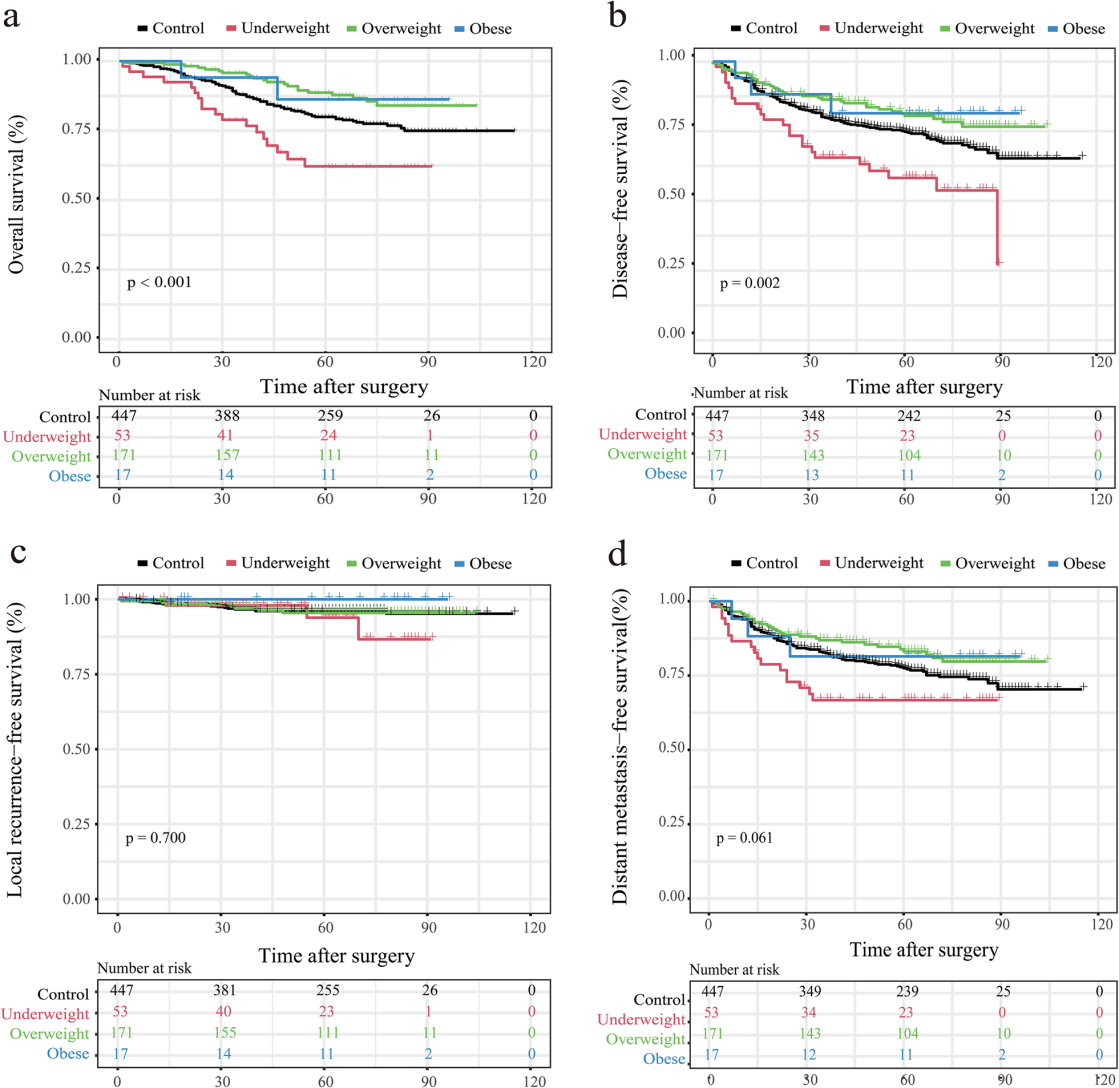
The cumulative **a**. overall survival (OS) rate, **b**. disease-free survival (DFS) rate, **c**. local recurrence free survival rate, **d**. distant metastasis free survival rate according to the BMI of all included rectal cancer patients (underweight, BMI < 18.5 kg/m^2^; control, 18.5 to < 25.0 kg/m^2^; overweight, 25.0 to < 30.0 kg/m^2^; obese, BMI ≥ 30 kg/m^2^)

As shown in Fig. 1, the OS and DFS in the underweight group were significantly poorer than those of the control group. The OS and DFS in the overweight and obese groups tended to be better than those of the control group. Concerning local recurrence-free survival, there was no significant difference among these groups. The underweight group had a trend toward poorer distant metastasis-free survival than the control group.

**Fig. 2:**
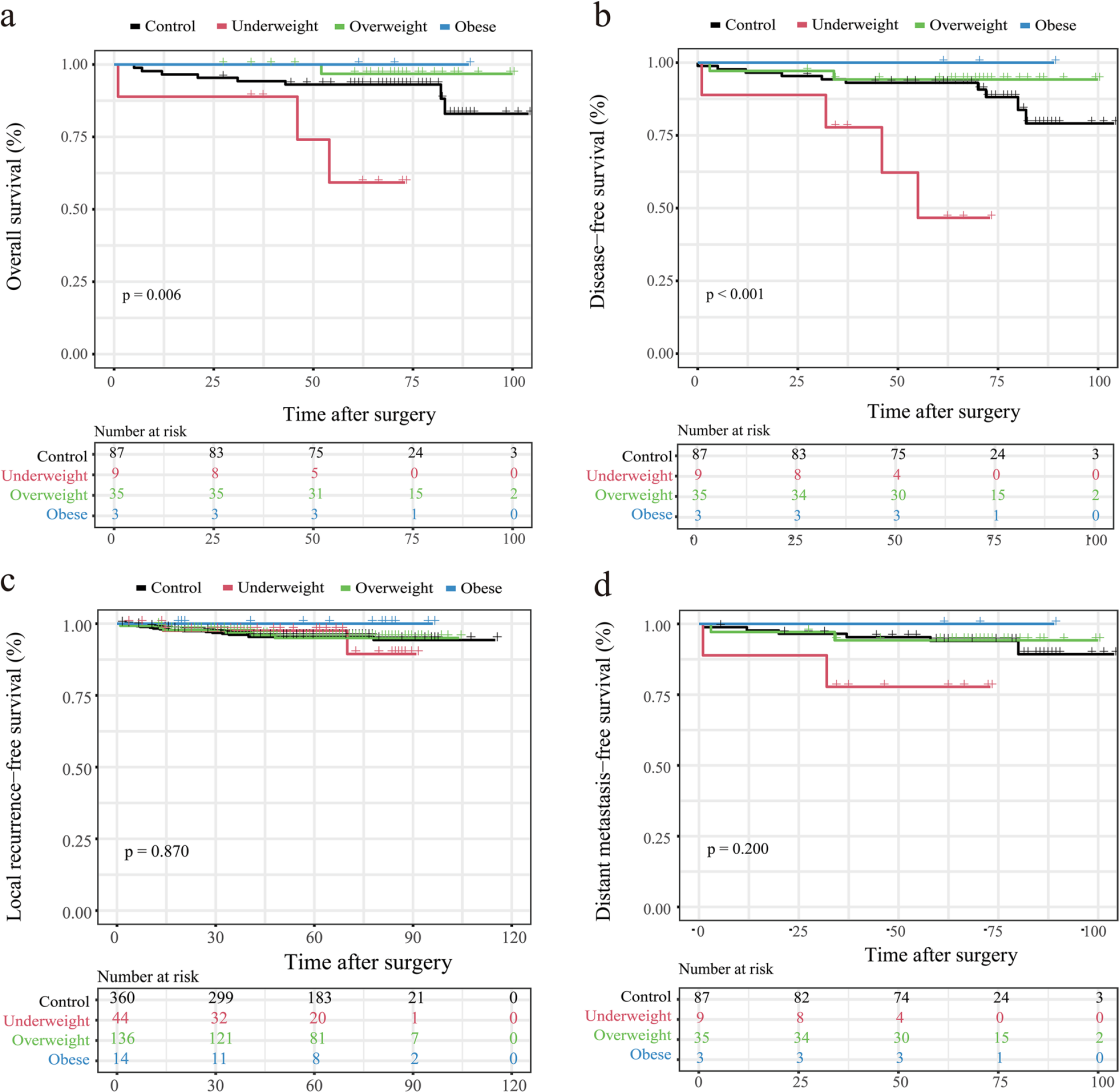
The cumulative **a**. overall survival (OS) rate, **b**. disease-free survival (DFS) rate, **c.** local recurrence free survival rate, **d**. distant metastasis free survival rate according to the BMI of cT1/2 rectal cancer patients (underweight, BMI < 18.5 kg/m^2^; control, 18.5 to < 25.0 kg/m^2^; overweight, 25.0 to < 30.0 kg/m^2^; obese, BMI ≥ 30 kg/m^2^)

**Fig. 3:**
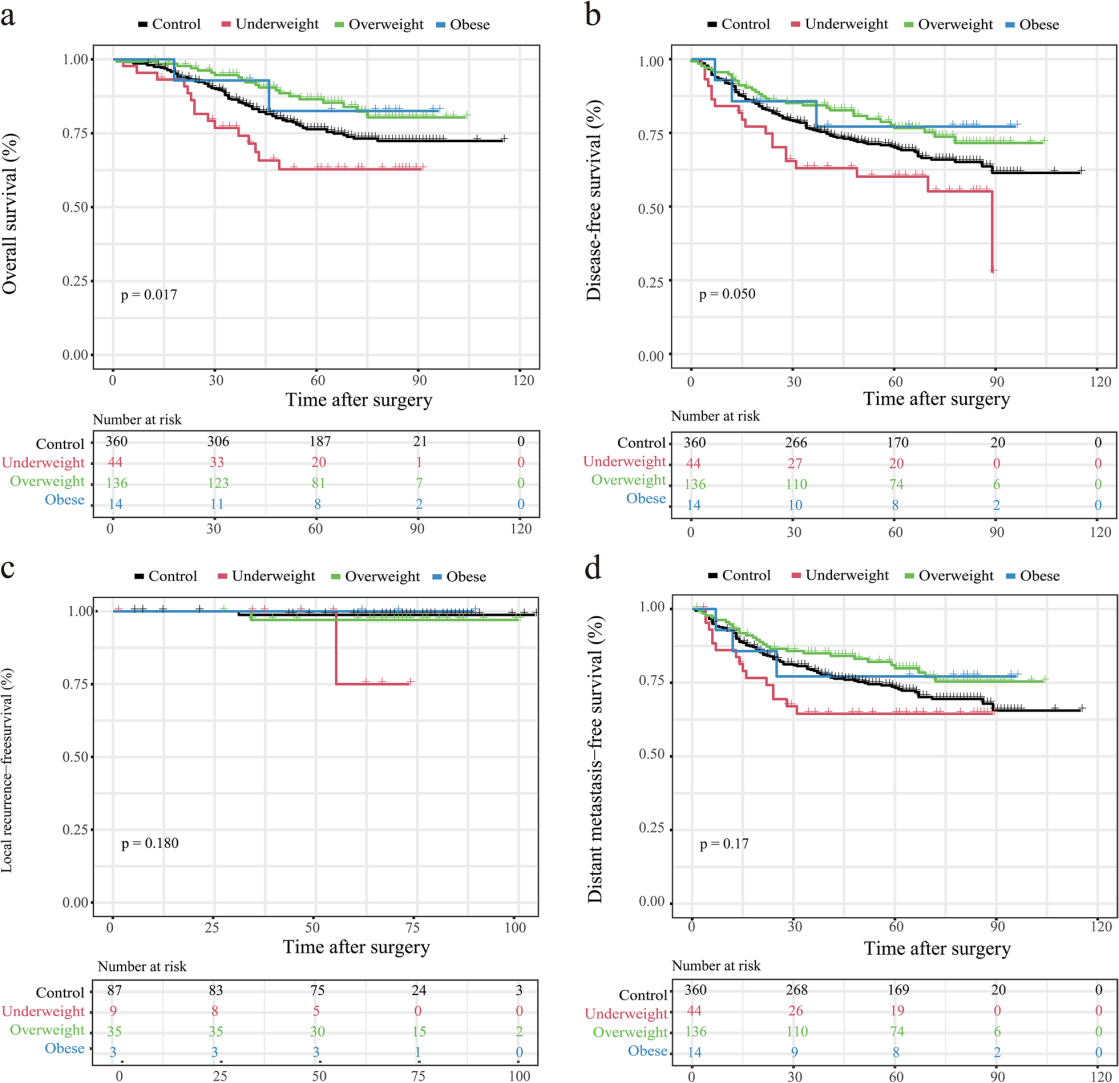
The cumulative **a**. overall survival (OS) rate, **b**. disease-free survival (DFS) rate, **c**. local recurrence free survival rate, **d**. distant metastasis free survival rate according to the BMI of cT3/4 rectal cancer patients (underweight, BMI < 18.5 kg/m^2^; control, 18.5 to < 25.0 kg/m^2^; overweight, 25.0 to < 30.0 kg/m^2^; obese, BMI ≥ 30 kg/m^2^)

We further subdivided the patients included into cT1/2 and cT3/4 groups due to the imbalance of cT stage among different groups, and subgroup analyses were performed. The results are presented in Figs. 2 and 3. In both cT1/2 and cT3/4 patients, the underweight group had significantly worse OS and DFS than the control group. However, there were no significant differences among the four groups regarding local recurrence-free survival and distant metastasis-free survival.

To reveal the independent risk factors for OS, DFS, local recurrence-free survival and distant metastasis-free survival, univariate analyses and multivariate analyses were performed. As shown in Table 2, age ≥ 65 years (HR 1.574; 95% CI 1.105–2.243; *p* = 0.012), non-sphincter-preserving surgery (HR 1.725; 95% CI 1.145–2.598; *p* = 0.009), pT stage (HR 1.666; 95% CI 1.112–2.495; *p* = 0.013) and pN stage (HR 2.218; 95% CI 1.545–3.186; *p* = 0.000) were independent risk factors for OS. Overweight (HR 0.534; 95% CI 0.332–0.860, *p* = 0.010) was an independent protective factor for OS and DFS (HR 0.675; 95% CI 0.461–0.989, *p* = 0.044). Non-sphincter preservation (HR 1.513; 95% CI 1.056–2.166; *p* = 0.024), pT stage (HR 1.557; 95% CI 1.108–2.189; *p* = 0.011) and pN stage (HR 2.582; 95% CI 1.900–3.210, *p* = 0.000) were independent risk factors for DFS. Of note, underweight was an independent risk factor for DFS (HR = 1.623; 95% CI 1.034–2.548; *p* = 0.035), and had a trend to be an independent risk factor for OS (HR 1.594; 95% 0.954–2.663; *p* = 0.075), although it did not reach statistical significance. Meanwhile, some independent risk factors for local recurrence-free survival and distant metastasis-free survival were shown in Table 3.

**Table 2:** Univariate and multivariate analyses of factors associated with the overall survival and Disease-free survival

Variables	Overall survival				Disease-free survival			
	Univariate analysis		Multivariate analysis		Univariate analysis		Multivariate analysis	
	HR (95% CI)	*p* value	HR (95% CI)	*p* value	HR (95% CI)	*p* value	HR (95% CI)	*p* value
Sex: female/male	1.061 (0.747, 1.509)	0.740			1.094 (0.810, 1.477)	0.558		
Age ≥ 65 years	1.110 (1.020, 2.032)	0.038	1.574 (1.105, 2.243)	**0.012**	1.090 (0.807, 1.472)	0.574		
Underweight (BMI < 18.5 kg/m^2^)	1.937 (1.168, 3.213)	0.010	1.594 (0.954, 2.663)	0.075	1.830 (1.172, 2.858)	0.008	1.623 (1.034, 2.548)	**0.035**
Overweight (BMI 25 to < 30 kg/m^2^)	0.567 (0.352, 0.911)	0.019	0.534 (0.332, 0.860)	**0.010**	0.691 (0.472, 1.010)	0.056	0.675 (0.461, 0.989)	**0.044**
Obese (BMI ≥ 30 kg/m^2^)	0.565 (0.139, 2.295)	0.425	0.643 (0.158, 2.620)	0.538	0.608 (0.193, 1.911)	0.394	0.690 (0.219, 2.176)	0.527
Tumor distance from AV ≤ 5 cm	1.257 (0.886, 1.782)	0.200			1.260 (0.935, 1.697)	0.129		
cT: 1,2/3,4	2.848 (1.573, 5.158)	0.001			2.998 (1.794, 5.009)	0.000		
cN: 0/1,2	1.592 (1.116, 2.270)	0.010			1.944 (1.422, 2.657)	0.000		
Preoperative short-course RT	1.177 (0.677, 2.047)	0.563			1.487 (0.959, 2.307)	0.076		
Preoperative long-course CRT	1.496 (1.011, 2.212)	0.044	1.411 (0.905, 2.201)	0.129	1.608 (1.154, 2.241)	0.005	1.511 (1.095, 2.085)	**0.012**
Preoperative chemotherapy	0.000 (0.000, 1.543 × 10^189^)	0.958			0.000 (0.000,2.652 × 10^158^)	0.951		
Adjuvant therapy	0.903 (0.641, 1.273)	0.560			1.171 (0.874, 1.567)	0.290		
Non-sphincter-preserving/Sphincter-preserving	1.688 (1.134, 2.515)	0.010	1.725 (1.145, 2.598)	**0.009**	1.480 (1.042, 2.103)	0.028	1.513 (1.056, 2.166)	**0.024**
pT: 0,1,2/3,4	2.197 (1.497, 3.222)	0.000	1.666 (1.112, 2.495)	**0.013**	2.141 (1.551, 2.954)	0.000	1.557 (1.108, 2.189)	**0.011**
pN: 0/1,2	2.359 (1.677, 3.319)	0.000	2.218 (1.545, 3.186)	**0.000**	2.875 (2.147, 3.849)	0.000	2.582 (1.900, 3.210)	**0.000**
Number of mesorectal lymph nodes harvested ≥ 12	0.762 (0.535, 1.084)	0.131			0.970 (0.723, 1.301)	0.838		

Statistically significant *p* values in multivariate analyses were shown in bold typeface. BMI, body mass index; AV, anal verge; RT, radiotherapy; CRT, chemoradiotherapy; HR, hazard ratio

**Table 3:** Univariate and multivariate analyses of factors associated with the local recurrence-free survival and distant metastasis-free survival

Variables	Local recurrence-free survival				Distant metastasis-free survival			
	Univariate analysis		Multivariate analysis		Univariate analysis		Multivariate analysis	
	HR (95% CI)	*p* value	HR (95% CI)	*p* value	HR (95% CI)	*p* value	HR (95% CI)	*p* value
Sex: female/male	0.883 (0.406, 1.924)	0.755			1.084 (0.782, 1.502)	0.628		
Age ≥ 65 years	0.558 (0.224, 1.389)	0.210			1.027 (0.739, 1.428)	0.875		
Underweight (BMI < 18.5 kg/m^2^)	1.771 (0.516, 6.082)	0.364	1.404 (0.402, 4.909)	0.595	1.370 (0.435, 4.319)	0.591	1.485 (0.887, 2.485)	0.133
Overweight (BMI 25 to < 30 kg/m^2^)	1.077 (0.443, 2.619)	0.870	1.187 (0.483, 2.916)	0.709	2.144 (0.628, 7.321)	0.223	0.730 (0.498, 1.128)	0.129
Obese (BMI ≥ 30 kg/m^2^)	0.000 (0.000, 3.568 × 10^168^)	0.981	0.000 (0.000, 3.572 × 10^168^)	0.984	0.988 (0.301, 3.239)	0.984	0.789 (0.250, 2.493)	0.686
Tumor distance from AV ≤ 5 cm	4.298 (1.968, 9.888)	0.001	5.556 (2.390, 12.919)	**0.000**	1.141 (0.823, 1.583)	0.429		
cT: 1,2/3,4	2.071 (0.621, 6.901)	0.236			4.112 (2.165, 7.810)	0.000		
cN: 0/1,2	1.405 (0.635, 3.106)	0.401			2.017 (1.431, 2.842)	0.000		
Preoperative short-course RT	3.203 (1.183, 8.673)	0.022	2.299 (0.905, 5.840)	0.080	1.531 (0.957, 2.449)	0.076		
Preoperative long-course CRT	2.181 (0.891, 5.336)	0.088			1.521 (1.061, 2.182)	0.023	1.476 (1.044, 2.088)	**0.028**
Preoperative chemotherapy	0.000 (0.000, 3.544 × 10^278^)	0.000	0.000 (0.000, 3.264 × 10^238^)	0.981	0.000 (0.000, 2.565 × 10^172^)	0.955		
Adjuvant therapy	1.094 (0.506, 2.363)	0.820			1.291 (0.940, 1.774)	0.115		
Non-sphincter-preserving/Sphincter-preserving	2.290 (0.995, 5.269)	0.051			1.296 (0.874, 1.922)	0.198		
pT: 0,1,2/3,4	6.420 (1.927, 21.388)	0.002	6.586 (1.908, 22.734)	**0.003**	2.208 (1.552, 3.140)	0.000	1.561 (1.076, 2.265)	**0.019**
pN: 0/1,2	3.165 (1.453, 6.895)	0.004	2.619 (1.168, 5.873)	**0.019**	3.090 (2.247, 4.251)	0.000	2.716 (1.943, 3.795)	**0.000**
Number of mesorectal lymph nodes harvested ≥ 12	0.381 (0.153, 0.949)	0.038	0.368 (0.146, 0.930)	**0.035**	1.078 (0.784, 1.481)	0.644		

Statistically significant *p* values in multivariate analyses were shown in bold typeface. BMI, body mass index; AV, anal verge; RT, radiotherapy; CRT, chemoradiotherapy; HR, hazard ratio

### Establishment and validation of nomograms

**Fig. 4:**
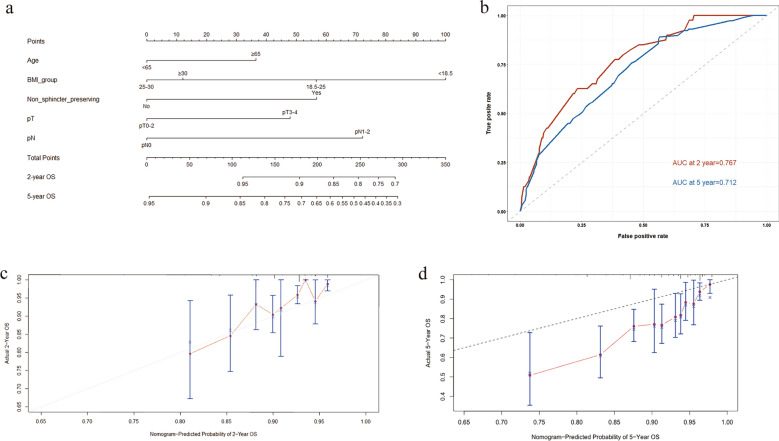
**a**. Nomogram for predicting 2-year OS and 5-year OS of rectal cancer patients. **b**. Receiver operating characteristic (ROC) curve based on the constructed nomogram. Calibration curve of the nomogram on **c**. 2-year OS and **d**. 5-year OS

To quantitatively predict the OS of these patients, a nomogram for predicting 2-year OS and 5-year OS was established (Fig. 4a). Age, BMI, pT, pN and nonsphincter-preserving surgery were included in the nomogram. The model performance was evaluated by the concordance index (C-index), Receiver operating characteristic (ROC) curves and the calibration curves[22–24]. The C-index of this nomogram was 0.698. ROC curves were further drawn to reveal the predictive power (Fig. 4b). The area under curve (AUC) was 0.767 (2-year OS) and 0.712 (5-year OS), respectively. The calibration curves revealed relatively good agreement between the predicted and actual probabilities for both 2-year OS and 5-year OS (Fig. 4c, d).

**Fig. 5:**
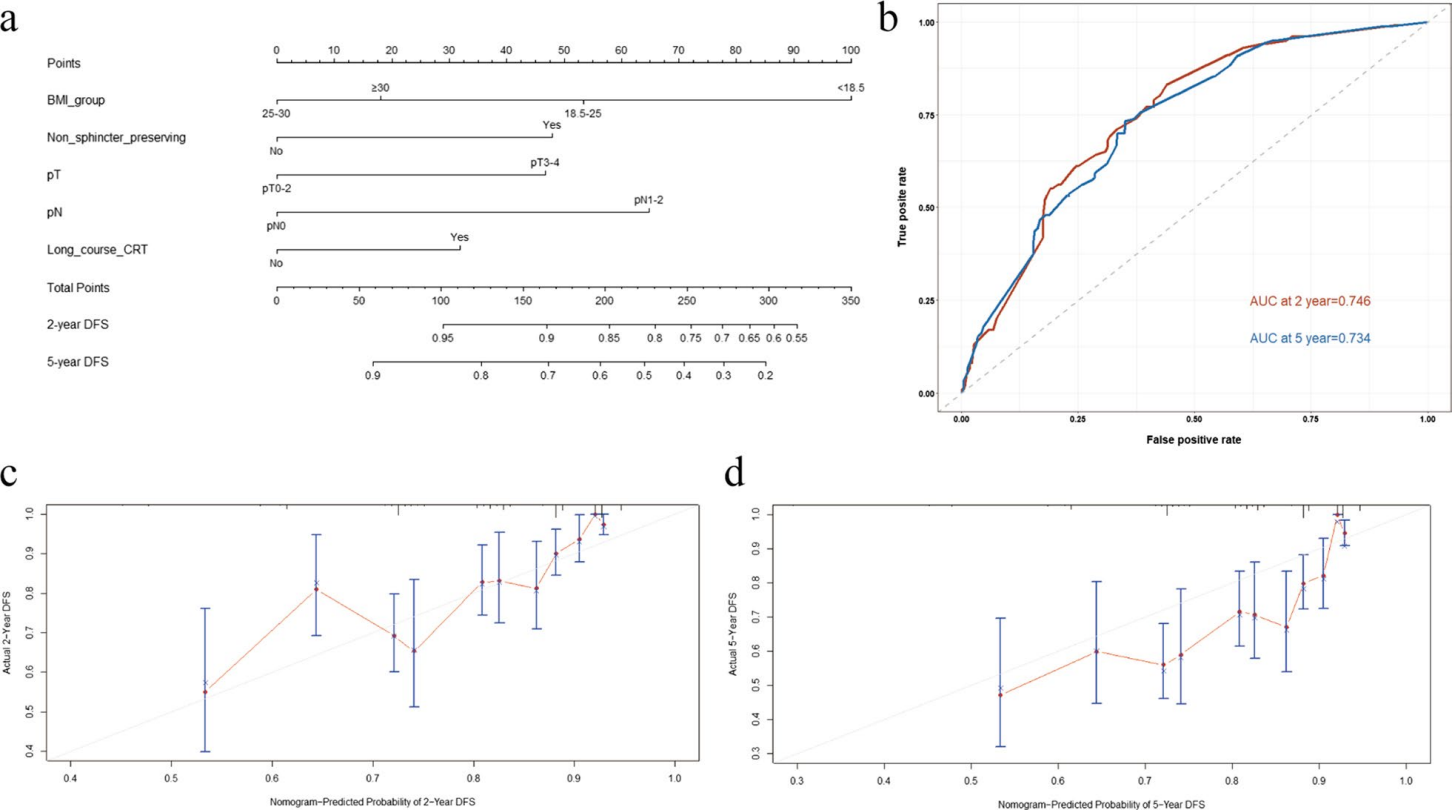
**a**. Nomogram for predicting 2-year DFS and 5-year DFS of rectal cancer patients. **b**. Receiver operating characteristic (ROC) curve based on the constructed nomogram. Calibration curve of the nomogram on **c**. 2-year DFS and **d**. 5-year DFS

Likewise, a nomogram for predicting 2-year DFS and 5-year DFS was created (Fig. 5a). Age, BMI, pT, pN, nonsphincter-preserving surgery and long-term CRT were included in this nomogram. The C-index was 0.691. ROC curves were drawn and shown in Fig. 5b. The AUC was 0.746 (2-year DFS) and 0.734 (5-year DFS), respectively. The calibration curves revealed relatively good agreement between the predicted and actual probabilities for both 2-year DFS and 5-year DFS (Fig. 5c, d).

## Discussion

In this study, we evaluated the effect of BMI on the long-term outcome of rectal cancer after radical surgery. We found that underweight was associated with significantly poorer OS and DFS. Univariate and multivariate analyses showed that overweight was an independent protective factor for OS and DFS. Underweight was an independent risk factor for DFS and had a trend to be an independent risk factor for OS.

According to the World Health Organization (WHO), over 1.9 billion adults were overweight in 2016, including 650 million obese adults [20]. High BMI was reported to be associated with an increased risk of a variety of tumors, including rectal cancer [25, 26]. A systematic review including 56 observational studies demonstrated that increased BMI led to higher morbidity of colorectal cancer. They further revealed that every 5 kg/m^2^ increase in BMI is associated with an 18% increase in risk [27]. Therefore, increased BMI is associated with a higher incidence of rectal cancer. However, whether an increased BMI will lead to a worse prognosis is still controversial.

Mayo Clinic published a large-sample study, which included 3,799 colorectal patients, demonstrating that an increased BMI caused a significantly better OS [15]. In detail, the 5-year OS rate for underweight was 33%, which was much lower than 56% for normal weight. The 5-year OS rates of overweight and obese patients were 60 and 65%, respectively. Another study, which included only local advanced rectal cancer (LARC) patients, stated that underweight was an independent risk factor for OS and cancer-specific survival (CSS). Additionally, one study reported that BMI ≥ 25 was significantly correlated with greater OS compared to BMI < 25 [12]. Although focusing on different colorectal cancer patients, these results were similar to our results. In the current study, with stage I/II/III rectal cancer patients included, the 5-year OS rates of the underweight, control, overweight and obese groups were 62.2%, 79.2%, 88.7% and 86.3%, respectively. We proposed that the inclusion of stage I patients and the fact that a substantial proportion of our patients received nCRT (42.4%) led to a relatively higher OS rate. The relatively small number of patients in the obese group might partly cause the comparable OS without a significant difference between the overweight and obese groups.

Nevertheless, some studies have reported the opposite results. A study showed that the LR rate increased with an increased BMI in lower rectal cancer patients (located within 10 cm from the anal verge) [9]. However, these patients included underwent surgery between 1995 and 2003. In our opinion, as surgeons improve their surgical skills in dealing with overweight/obese patients, there would not be a significant difference in the LR rate. Similar to the results observed in our study, there was no significant difference in local recurrence-free survival. Another study pointed out that BMI ≥ 25.6 was associated with more anastomotic recurrences in stage II/III patients treated with radical surgery and perioperative radiotherapy [11]. However, they did not compare OS or CSS in this study. Therefore, sincemore anastomotic recurrences did not necessarily cause worse OS, we cannot conclude that higher BMI led to poorer survival.

Regarding the imbalance of cT stage in the clinicopathological characteristics among different BMI groups. Patients were subdivided into cT1/2 and cT3/4, and subgroup analyses were further performed. The results were consistent with those of pooled analyses. In both cT1/2 and cT3/4 patients, the underweight group had significantly worse OS and DFS than the control group. However, a study reported that the impact of BMI on OS was different across the stages of rectal cancer. In detail, the OS of obese patients is comparable to that of normal patients in stage 1/2. In stage 3/4, the underweight group revealed a significantly worse OS[15]. In fact, underweight or malnutrition commonly occurs during nCRT in rectal cancer patients. The BMI of this study was measured prior to rectal cancer diagnosis. In addition, they further demonstrated that a postdiagnosis BMI drop of more than 10% from a prediagnosis could predict worse OS. In our study, we only recorded BMI before surgery. Therefore, the effect of changes in BMI could not be assessed, which was a limitation of our study.

Multivariate analyses were performed to reveal the independent risk factors associated with long-term outcomes. Overweight was an independent protective factor for OS and tended to be an independent protective factor for DFS. Underweight was an independent risk factor for OS and DFS. Unlike our study, another study revealed that both underweight (BMI < 20 kg/m^2^) [HR 4.070, *p* = 0.002] and overweight (25–26.9 kg/m^2^) [HR 2.077, *p* = 0.010] were risk factors for OS[17]. Different BMI groupings may lead to differences. Apart from BMI, pT stage and pN stage, we also found that age ≥ 65 years and non-sphincter preserving surgery were independent risk factors for OS. Non-sphincter preservation and preoperative long-course CRT were independent risk factors for DFS. Nomograms incorporating BMI and these prognostic factors were established to quantitatively predict the 2-year and 5-year OS and DFS. ROC curves were further drawn and showed that AUC was 0.767 (2-year OS), 0.712 (5-year OS), 0.746 (2-year DFS) and 0.734 (5-year DFS), respectively. A study built a nomogram, which was based on BMI, neural invasion, pre-CA199, ypStage, and adjuvant chemotherapy, revealing a C-index of 0.837[14]. But they did not draw ROC curves to assess the predictive power. Actually, it is quite difficult to predict long-term survival due to a lot of other contributing factors. Based on preoperative CEA and several clinicopathological factors (age, gender, cT, cN, grade, perineural involvement, tumor deposits), another study established a nomogram to predict OS and found the C-index was higher than that of the TNM staging system (0.71 vs 0.58) [28]. Therefore, with a C-index of 0.698 and 0.691 for predicting OS and DFS, our nomograms revealed relatively good predictive power.

As a study to assess the prognostic value of BMI in rectal cancer patients, we did not analyze the relationship of BMI with postoperative complications, which is a limitation of this study. A meta-analysis including 2519 patients who received gastrointestinal surgery across 127 centers, found that obesity was not related to 30-day postoperative major complications [29]. However, this study included all patients who underwent gastrointestinal surgery (both elective and emergency), which is quite different from the patients in our study. Another study focused on the risk factors for anastomotic leakage in rectal cancer patients who underwent anterior resection surgery. The results showed that BMI was one of the independent risk factors for 30-day anastomotic leakage [30]. Prospective studies are needed to further elucidate the relationship of BMI with postoperative complications in rectal cancer patients.

The following are some limitations of this study. First, it is a retrospective study, and selection bias cannot be completely avoided. Nevertheless, subgroup analyses and multivariate analyses were conducted to reduce the effect of confounders. Second, the relatively small sample sizes in the overweight and obese groups made it difficult to find significant differences between these two groups. Third, we only recorded BMI before surgery, making it impossible to assess the impact of changes in BMI. Fourth, as we mentioned above, the relationship of BMI with postoperative complications was elucidated.

## Conclusion

For rectal cancer patients after radical surgery, overweight was an independent protective factor for OS and DFS. Underweight was an independent risk factor for DFS and had a trend to be an independent risk factor for OS. Nomograms incorporating BMI and other prognostic factors could be helpful to predict long-term outcome.

## Supplementary Information

**Additional file 1.** The STROBE Statement-checklist of items that should be addressed in reports of observational studies.

## Data Availability

All data generated or analysed during this study are included in this published article. For further inquiries, please contact the corresponding author (wangziqiang@scu.edu.cn).
