# Correction: The effect of BMI on long-term outcome in patients with rectal cancer and establishment of a nomogram prediction model

**DOI:** 10.1186/s12876-026-05104-w

**Published:** 2026-08-10

**Authors:** Yang Zhang, Xuyang Yang, Zixuan Zhuang, Mingtian Wei, Wenjian Meng, Xiangbing Deng, Ziqiang Wang

**Affiliations:** Colorectal Cancer Center, Department of General Surgery, West China Hospital, Sichuan University, No. 37 Guo Xue Alley, Chengdu, 610041 China

**Correction: BMC Gastroenterol 23, 5 (2023)**

**https://doi.org/10.1186/s12876-023-02638-1**

Following publication of the original article [1] it was reported there was an error in Fig. 4, namely that panels 4 C and 4D were duplicates.

The incorrect Fig. 4 was:

**Figure:**
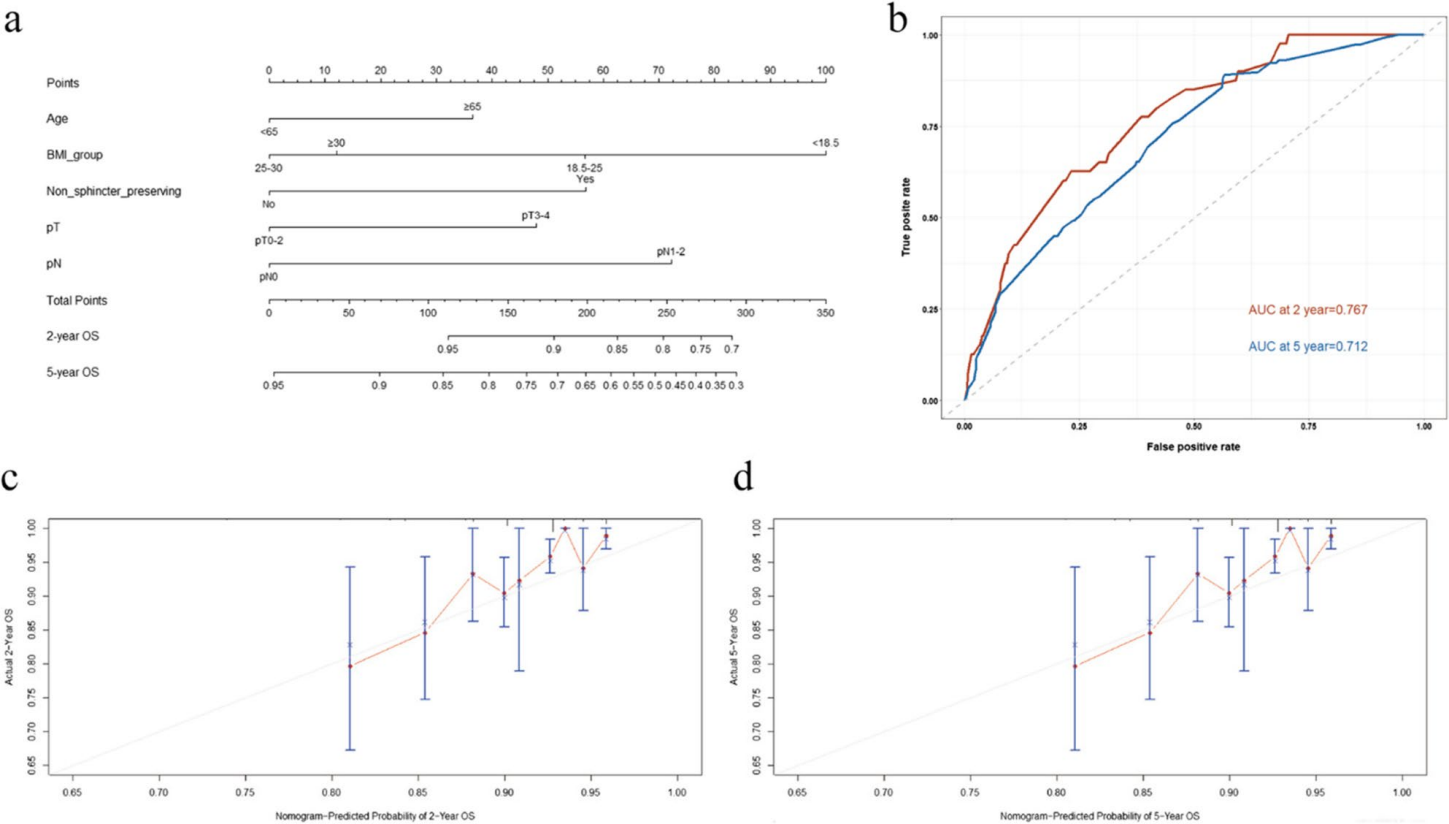

The correct Fig. 4 is:

**Figure:**
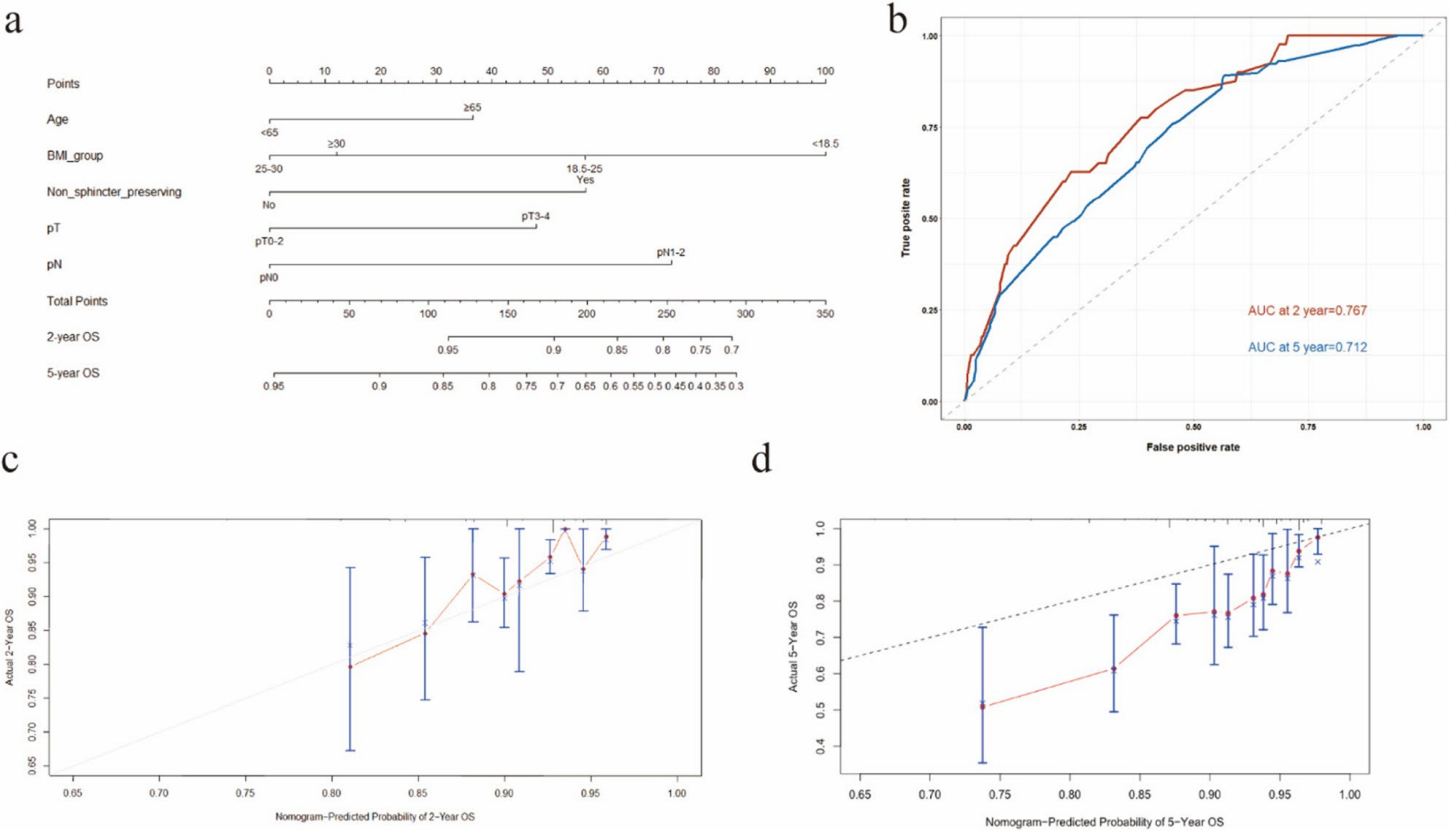

The original article has been updated.
