# Supplementary material for: The effect of BMI on long-term outcome in patients with rectal cancer and establishment of a nomogram prediction model
Source: BMC Gastroenterol. 2023 Jan 9;23:5. doi: 10.1186/s12876-023-02638-1 (PMC9830815; doi:10.1186/s12876-023-02638-1)
Supplement: Supplementary file 1 — Additional file 1. The STROBE Statement-checklist of items that should be addressed in reports of observational studies. [file 12876_2023_2638_MOESM1_ESM.doc]

**The STROBE Statement-checklist of items that should be addressed in reports of observational studies.**

|  | Item No | Recommendation |
| --- | --- | --- |
| **Title and abstract** | 1 | (*a*) Indicate the study’s design with a commonly used term in the title or the abstract  Title Page |
|  |  | (*b*) Provide in the abstract an informative and balanced summary of what was done and what was found  Abstract |
| Introduction | | |
| Background/rationale | 2 | Explain the scientific background and rationale for the investigation being reported  Introduction paragraphs 1-2 |
| Objectives | 3 | State specific objectives, including any prespecified hypotheses  Introduction paragraph 3 |
| Methods | | |
| Study design | 4 | Present key elements of study design early in the paper  Abstract, Materials and methods paragraph 1 |
| Setting | 5 | Describe the setting, locations, and relevant dates, including periods of recruitment, exposure, follow-up, and data collection  Materials and methods paragraph 1-4 |
| Participants | 6 | (*a*) Give the eligibility criteria, and the sources and methods of selection of participants. Describe methods of follow-up  Materials and methods paragraph 1-4 |
|  |  | (*b*) For matched studies, give matching criteria and number of exposed and unexposed  Not applicable |
| Variables | 7 | Clearly define all outcomes, exposures, predictors, potential confounders, and effect modifiers. Give diagnostic criteria, if applicable  Materials and methods paragraph 2 |
| Data sources/ measurement | 8* | For each variable of interest, give sources of data and details of methods of assessment (measurement). Describe comparability of assessment methods if there is more than one group  Materials and methods "Statistical analysis" |
| Bias | 9 | Describe any efforts to address potential sources of bias  Materials and methods "Statistical analysis" |
| Study size | 10 | Explain how the study size was arrived at  Not applicable |
| Quantitative variables | 11 | Explain how quantitative variables were handled in the analyses. If applicable, describe which groupings were chosen and why  Materials and methods "Statistical analysis" |
| Statistical methods | 12 | (*a*) Describe all statistical methods, including those used to control for confounding  Materials and methods "Statistical analysis" |
|  |  | (*b*) Describe any methods used to examine subgroups and interactions  Materials and methods "Statistical analysis" |
|  |  | (*c*) Explain how missing data were addressed  Not applicable |
|  |  | (*d*) If applicable, explain how loss to follow-up was addressed  Not applicable |
|  |  | (*e*) Describe any sensitivity analyses  Not applicable |
| Results | | |
| Participants | 13* | (a) Report numbers of individuals at each stage of study—eg numbers potentially eligible, examined for eligibility, confirmed eligible, included in the study, completing follow-up, and analysed  Results paragraph 1 |
|  |  | (b) Give reasons for non-participation at each stage  Not applicable |
|  |  | (c) Consider use of a flow diagram  Not applicable |
| Descriptive data | 14* | (a) Give characteristics of study participants (eg demographic, clinical, social) and information on exposures and potential confounders  Results paragraph 1 |
|  |  | (b) Indicate number of participants with missing data for each variable of interest  Not applicable |
|  |  | (c) Summarise follow-up time (eg, average and total amount)  Results paragraph 2 |
| Outcome data | 15* | Report numbers of outcome events or summary measures over time  Results paragraph 2-5 |
| Main results | 16 | (*a*) Give unadjusted estimates and, if applicable, confounder-adjusted estimates and their precision (eg, 95% confidence interval). Make clear which confounders were adjusted for and why they were included  Results paragraphs 2-5 |
|  |  | (*b*) Report category boundaries when continuous variables were categorized  Results paragraphs 1 |
|  |  | (*c*) If relevant, consider translating estimates of relative risk into absolute risk for a meaningful time period  Not applicable |
| Other analyses | 17 | Report other analyses done—eg analyses of subgroups and interactions, and sensitivity analyses  Results paragraph 4-7 |
| Discussion | | |
| Key results | 18 | Summarise key results with reference to study objectives  Discussion paragraph 1 |
| Limitations | 19 | Discuss limitations of the study, taking into account sources of potential bias or imprecision. Discuss both direction and magnitude of any potential bias  Discussion paragraph 8 |
| Interpretation | 20 | Give a cautious overall interpretation of results considering objectives, limitations, multiplicity of analyses, results from similar studies, and other relevant evidence  Discussion paragraphs 3-6 |
| Generalisability | 21 | Discuss the generalisability (external validity) of the study results  conclusion paragraph 1 |
| Other information | | |
| Funding | 22 | Give the source of funding and the role of the funders for the present study and, if applicable, for the original study on which the present article is based  Funding |

*Give information separately for exposed and unexposed groups.

**Note:** An Explanation and Elaboration article discusses each checklist item and gives methodological background and published examples of transparent reporting. The STROBE checklist is best used in conjunction with this article (freely available on the Web sites of PLoS Medicine at http://www.plosmedicine.org/, Annals of Internal Medicine at http://www.annals.org/, and Epidemiology at http://www.epidem.com/). Information on the STROBE Initiative is available at http://www.strobe-statement.org.
